# Ten years of inhibition revisited

**DOI:** 10.3389/fnhum.2014.00329

**Published:** 2014-05-21

**Authors:** Diane Swick, Christopher H. Chatham

**Affiliations:** ^1^Research Service, VA Northern California Health Care SystemMartinez, CA, USA; ^2^Department of Neurology, University of CaliforniaDavis, CA, USA; ^3^Cognitive, Linguistic and Psychological Sciences, Brown UniversityProvidence, RI, USA

**Keywords:** response inhibition, inhibitory control, context monitoring, right inferior frontal gyrus, left inferior frontal gyrus

In their 2004 Trends in Cognitive Sciences review of inhibition and the right inferior frontal cortex (rIFC), Aron et al. (henceforth AR&P) boldly claimed that “inhibition is localized to right IFG alone” (Aron et al., 2004). Ten years later, the authors have updated their theory to include one or more fronto–basal–ganglia networks along with rIFC, and to characterize the function of rIFC as a “brake” that can completely stop or otherwise slow behavioral responses (Aron et al., 2014). AR&P also examined (and dismissed) two main lines of contrary evidence that question whether the rIFC is the critical locus for inhibition, and whether inhibition is the primary function of rIFC. The revisions can account for some findings outside AR&P's initial conception of inhibitory control. However, we maintain that the revised theory is potentially unfalsifiable and still strongly challenged by prior evidence. Below, we discuss some of the data that pose greater difficulties for the hypothesis than AR&P have acknowledged.

AR&P first address critics of the rIFC specificity view. Based on their prior lesion results, AR&P argue that right and (not left) IFC is critical for inhibition in the Stop-Signal task (Aron et al., 2003). They discount key findings from patients with left IFC lesions in the Go/NoGo task (Swick et al., 2008) by arguing that deficits in non-inhibitory decision processes can account for worse performance when Go and NoGo trials are equiprobable. This rebuttal misses the main point: left IFC damage disproportionately impaired inhibition in the condition with infrequent NoGo trials, when inhibitory demands were greatest (Swick et al., 2008). This, along with the finding that omission errors on Go trials were not increased, contradicts AR&P's claim that left IFC damage differentially impacts the decision to go. A further speculation was that lesions of the insula reduced the degree of autonomic arousal related to stopping, thereby accounting for slower RTs in the patients. This idea was not supported by the data, as there was no relationship between RT and the amount of insula damage.

In addition, work uncited by AR&P (Krämer et al., 2013) failed to replicate the critical rIFC lesion results in the Stop-Signal task (Aron et al., 2003). This same study did replicate an important role for left IFC in inhibition in the Go/NoGo task (Krämer et al., 2013). Conclusions drawn from “virtual lesion” data are also ignored, including a transcranial magnetic stimulation (TMS) study in which stopping deficits were taken to reflect more general deficits in action programming (Verbruggen et al., 2010).

Just as these challenging lesion data are left unaddressed, AR&P also overlook the stronger challenges posed by neuroimaging. For example, AR&P reaffirm classic views of inhibition as a means for goal-driven control, but fail to explain why stopping/braking should occur even when it runs contrary to task goals; why rIFC is more strongly recruited in those situations than during the Stop task itself; or why rIFC recruitment is sustained even when subjects must always produce a “go” response, and proactive inhibitory control is unnecessary (Chatham et al., 2012). AR&P argue that rIFC BOLD could reflect stopping that occurs too late to affect behavior, but the positive correlation between rIFC BOLD and stopping speed (Whelan et al., 2012) renders this argument incapable of explaining the data.

The use of undetectable effects as an explanatory construct also raises the issue of falsifiability. While AR&P propose falsification criteria, they are ill-posed. For example, could one ever prove a task lacks all inhibitory demands, if these are imposed even by tasks that never require withholding a prepotent response? Similarly, could one prove a lack of damage to “connections” in a real frontal patient or TMS subject?

These criteria contrast with the weaker conditions used for “refuting” alternative perspectives, such as those that emphasize context monitoring instead of braking (Chatham et al., 2012). For example, AR&P claim to refute monitoring accounts by noting that rIFC electrocorticographic (ECoG) activity is more tightly linked with responses than stop signals, but this relationship held for only a minority of subjects (Swann et al., 2009). And leaving aside that monitoring is most critical in the midst of ongoing behavior (as shown by Chevalier et al., 2014), stop signals were not actually presented on the trials in question. AR&P also argue for the anatomical specificity of ECoG stopping responses in rIFC, when in fact similar activity patterns were recorded outside rIFC (Swann et al., 2009).

More broadly, AR&P continue to interpret many results as though they reflect an act of inhibitory control, but elsewhere acknowledge that stopping may be inextricably linked with salience detection (Wessel and Aron, 2013). This alternative is particularly difficult to eliminate with direct electrical stimulation (DES) (as used in Wessel et al., 2013), given that whole-field visual hallucinations can result from DES to IFC (either left or right; Blanke et al., 2000; Vignal et al., 2000). Even subthreshold effects of this kind could disrupt performance when subjects are looking for salient visual stimuli (Borchers et al., 2012).

To adequately evaluate whether the rIFC is differentially involved in stopping, rIFC must be compared with co-activated regions (Swick et al., 2011) (e.g., left IFC) both during conditions that require stopping and those that don't, but are otherwise matched for saliency, behavioral-relevance, error likelihood and awareness, and other attentional demands. If the predicted dissociations are not assessed in this way (as they often have not been), are only inconsistently obtained, or are impossible to test, then the claims should be broadened to those supported by evidence, and terminology changed accordingly (e.g., from stopping to monitoring/stopping).

Although we disagree that AR&P's reformulated hypothesis is a viable account of the extant data, we also wish to mention their impressive successes. Their work remains highly influential, inspiring vigorous debate and constituting a major success in linking brain and behavior. We credit AR&P and colleagues with these significant achievements, even if we continue to disagree on the specificity of rIFC's role in behavioral control.

## Acknowledgment

We wish to thank David Badre, Yuko Munakata, Thomas Wiecki, and Ashley Wu for their helpful comments and suggestions.

### Conflict of interest statement

The authors declare that the research was conducted in the absence of any commercial or financial relationships that could be construed as a potential conflict of interest.
